# Supplementary material for: MetaRibo-Seq measures translation in microbiomes
Source: Nat Commun. 2020 Jun 29;11:3268. doi: 10.1038/s41467-020-17081-z (PMC7324362; doi:10.1038/s41467-020-17081-z)
Supplement: Supplementary file 10 — Supplementary Data 7 [file 41467_2020_17081_MOESM10_ESM.zip › File2/Confidence_High_Taxonomy/141214_out.krona.html]

Javascript must be enabled to view this page.

members
magnitude
magnitudeUnassigned
count
unassigned
taxon
rank

141214\_out

155

2
155
superkingdom

phylum
155
976

class
155
200643

171549
155
order

815
2
family

2
genus
816


SRS078242\_contig\_number\_11165
1
species
310297


SRS016095\_contig\_number\_6201
1
species
329854

1853231
152
family


SRS013965\_contig\_number\_20705SRS017103\_contig\_number\_41515SRS021948\_contig\_number\_12555SRS024331\_contig\_number\_1784SRS050299\_contig\_number\_6766SRS050941\_contig\_number\_12623SRS077335\_contig\_number\_contig-100\_4334.4334SRS143523\_contig\_number\_12144SRS147425\_contig\_number\_2727SRS148874\_contig\_number\_13256SRS893231\_contig\_number\_2881SRS893252\_contig\_number\_19074
152
genus
12
283168

species
1

SRS024132\_contig\_number\_9867
626933

species

SRS015264\_contig\_number\_18808SRS056259\_contig\_number\_26200SRS062654\_contig\_number\_27552
3
1263090

28118
species

SRS011084\_contig\_number\_28521SRS011134\_contig\_number\_39001SRS011239\_contig\_number\_23669SRS011302\_contig\_number\_17301SRS012849\_contig\_number\_contig-100\_1169.190691SRS012969\_contig\_number\_34576SRS013098\_contig\_number\_42501SRS013521\_contig\_number\_contig-100\_166.60955SRS014459\_contig\_number\_6642SRS014683\_contig\_number\_13705SRS015065\_contig\_number\_16469SRS015431\_contig\_number\_63137SRS015579\_contig\_number\_6997SRS015663\_contig\_number\_14297SRS015782\_contig\_number\_39239SRS016018\_contig\_number\_11873SRS016056\_contig\_number\_7273SRS016335\_contig\_number\_11121SRS016954\_contig\_number\_22740SRS017191\_contig\_number\_18603SRS017247\_contig\_number\_8165SRS017433\_contig\_number\_11502SRS017521\_contig\_number\_30892SRS018133\_contig\_number\_13039SRS018623\_contig\_number\_contig-100\_391.129527SRS018817\_contig\_number\_14323SRS018836\_contig\_number\_contig-100\_438.155925SRS018888\_contig\_number\_10301SRS019030\_contig\_number\_12157SRS019068\_contig\_number\_52845SRS019161\_contig\_number\_27385SRS019381\_contig\_number\_19642SRS019397\_contig\_number\_3174SRS019601\_contig\_number\_13324SRS020233\_contig\_number\_41963SRS020271\_contig\_number\_600SRS020328\_contig\_number\_15842SRS020394\_contig\_number\_13286SRS021219\_contig\_number\_2153SRS021484\_contig\_number\_30895SRS022609\_contig\_number\_45997SRS024075\_contig\_number\_4466SRS024492\_contig\_number\_14207SRS024625\_contig\_number\_contig-100\_153.58078SRS043001\_contig\_number\_6121SRS043701\_contig\_number\_14660SRS043768\_contig\_number\_30124SRS043841\_contig\_number\_9468SRS044535\_contig\_number\_20641SRS045244\_contig\_number\_79SRS045713\_contig\_number\_3081SRS046502\_contig\_number\_contig-100\_575.576SRS046717\_contig\_number\_6817SRS047044\_contig\_number\_9266SRS047433\_contig\_number\_14464SRS048060\_contig\_number\_1461SRS048164\_contig\_number\_23918SRS048870\_contig\_number\_2669SRS049402\_contig\_number\_2367SRS049446\_contig\_number\_11157SRS049773\_contig\_number\_19615SRS049995\_contig\_number\_17472SRS050026\_contig\_number\_4524SRS050422\_contig\_number\_18570SRS051031\_contig\_number\_30180SRS051610\_contig\_number\_10672SRS051610\_contig\_number\_contig-100\_421.70059SRS051882\_contig\_number\_19380SRS053214\_contig\_number\_14469SRS053398\_contig\_number\_15393SRS054905\_contig\_number\_21909SRS055017\_contig\_number\_19380SRS058770\_contig\_number\_14369SRS063040\_contig\_number\_32390SRS063190\_contig\_number\_14390SRS063370\_contig\_number\_12241SRS063518\_contig\_number\_26994SRS063985\_contig\_number\_22983SRS064276\_contig\_number\_36128SRS064557\_contig\_number\_contig-100\_615.122143SRS064757\_contig\_number\_16148SRS065504\_contig\_number\_10949SRS076804\_contig\_number\_2912SRS077086\_contig\_number\_10792SRS077194\_contig\_number\_25727SRS077231\_contig\_number\_contig-100\_331.81811SRS077294\_contig\_number\_18233SRS077552\_contig\_number\_12981SRS077730\_contig\_number\_3476SRS077849\_contig\_number\_25950SRS078176\_contig\_number\_13972SRS078419\_contig\_number\_contig-100\_1615.213433SRS078665\_contig\_number\_6092SRS098571\_contig\_number\_45611SRS098644\_contig\_number\_31800SRS098827\_contig\_number\_3438SRS098881\_contig\_number\_18540SRS100021\_contig\_number\_10902SRS103987\_contig\_number\_30377SRS104036\_contig\_number\_467SRS1041037\_contig\_number\_2738SRS1041137\_contig\_number\_6207SRS104636\_contig\_number\_30542SRS104975\_contig\_number\_21252SRS105153\_contig\_number\_31064SRS140492\_contig\_number\_32073SRS140513\_contig\_number\_9741SRS142890\_contig\_number\_37965SRS143181\_contig\_number\_4173SRS143417\_contig\_number\_contig-100\_325.183442SRS143598\_contig\_number\_10735SRS143991\_contig\_number\_23601SRS144183\_contig\_number\_18210SRS144362\_contig\_number\_30490SRS144506\_contig\_number\_56666SRS146764\_contig\_number\_4996SRS147139\_contig\_number\_37049SRS147271\_contig\_number\_11025SRS147346\_contig\_number\_43648SRS147557\_contig\_number\_24140SRS148091\_contig\_number\_10672SRS148159\_contig\_number\_54743SRS148196\_contig\_number\_14702SRS148424\_contig\_number\_34519SRS148721\_contig\_number\_contig-100\_292.303611SRS149075\_contig\_number\_2550SRS893172\_contig\_number\_2631SRS893187\_contig\_number\_636SRS893341\_contig\_number\_13967SRS893378\_contig\_number\_contig-100\_262.113362SRS971275\_contig\_number\_7850
131

species

SRS055982\_contig\_number\_14444SRS077127\_contig\_number\_15625SRS077641\_contig\_number\_contig-100\_4035.112127SRS101433\_contig\_number\_12291SRS1041134\_contig\_number\_2887
5
1897010

family
1
171550

239759
genus
1

626932
1

SRS143876\_contig\_number\_27737
species
